# Supplementary material for: Expanding the Coverage of Metabolic Landscape in Cultivated Rice with Integrated Computational Approaches
Source: Genomics Proteomics Bioinformatics. 2021 Feb 23;20(4):702–14. doi: 10.1016/j.gpb.2020.06.018 (PMC9880819; doi:10.1016/j.gpb.2020.06.018)
Supplement: Supplementary Figure S1 — The performance evaluation of two annotation approaches A. Evaluating the performance of annotation with experimental mass spectra as reference. Query mass spectra were sampled from Metlin or Massbank, and spectral similarity was scored with NDP or INCOS algorithm. B. Evaluating the performance of annotation with in silico mass spectra as reference. Query mass spectra were sampled from Metlin or Massbank, and spectral similarity was scored by CFM-ID software [file mmc1.pdf]

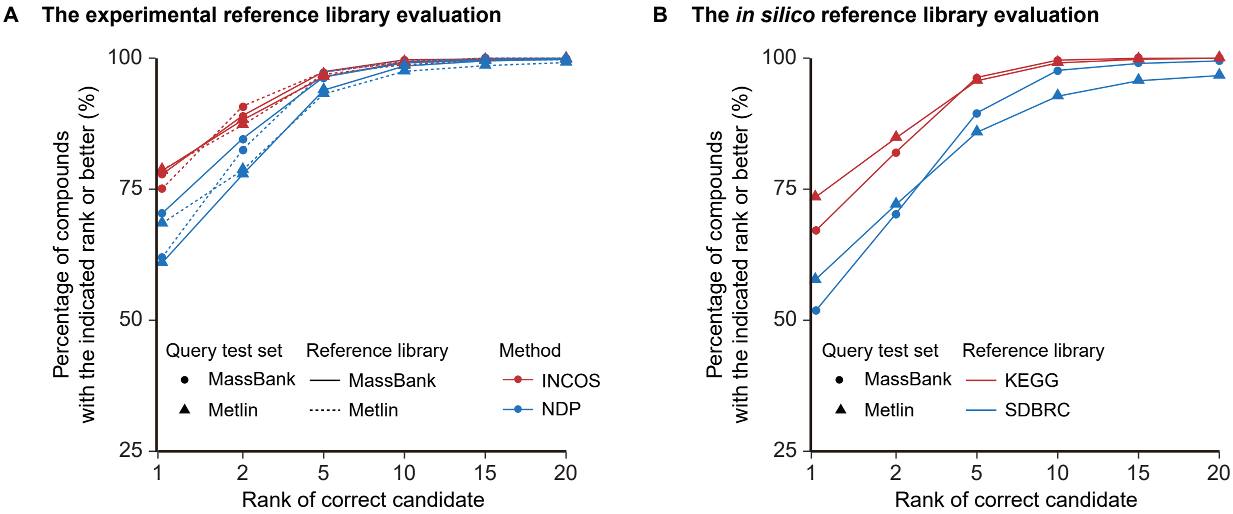
A The experimental reference library evaluation
B The in silico reference library evaluation
Percentage of compounds with the indicated rank or better (%)
Rank of correct candidate
100
75
50
25
1
2
5
10
15
20
Query test set
MassBank
Metlin
Reference library
MassBank
Metlin
Method
INCOS
NDP
KEGG
SDBRC
