# Supplementary material for: Expanding the Coverage of Metabolic Landscape in Cultivated Rice with Integrated Computational Approaches
Source: Genomics Proteomics Bioinformatics. 2021 Feb 23;20(4):702–14. doi: 10.1016/j.gpb.2020.06.018 (PMC9880819; doi:10.1016/j.gpb.2020.06.018)
Supplement: Supplementary Figure S2 — The workflow for MS2T library construction Sample preparation, metabolite extraction, UPLC-HRMS analysis, and mass spectral data processing were as described (see Materials and methods). UPLC-Q-Orbitrap-HRMS, ultra-performance liquid chromatography coupled with hybrid quadrupole-Orbitrap high-resolution mass spectrometry; MS2T, MS2 spectral tag [file mmc2.pdf]

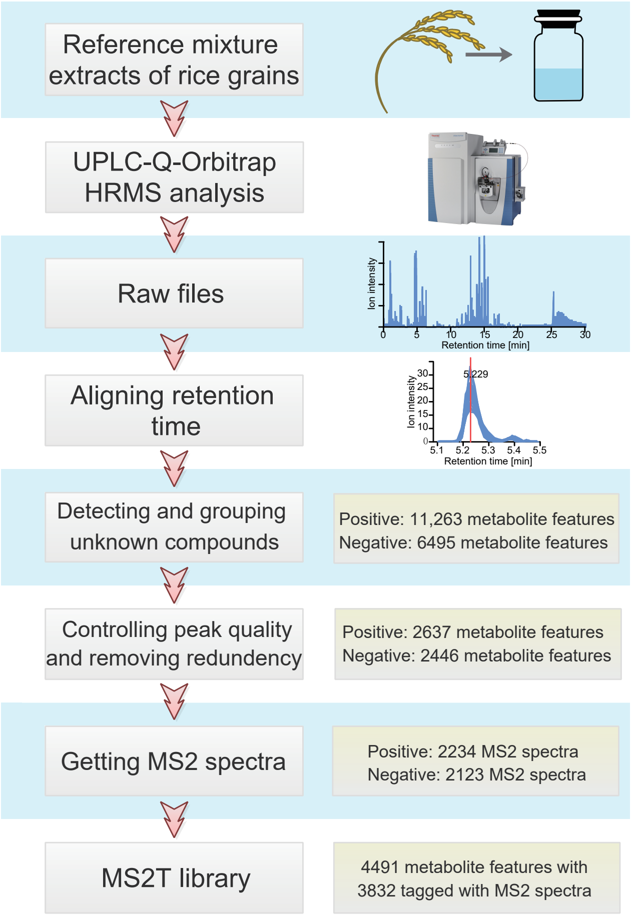
Reference mixture extracts of rice grains
UPLC-Q-Orbitrap HRMS analysis
Raw files
Ion intensity
0 5 10 15 20 25 30
Retention time [min]
Aligning retention time
Ion intensity
0 10 15 20 25 30
5.229
5.1 5.2 5.3 5.4 5.5
Retention time [min]
Detecting and grouping unknown compounds
Positive: 11,263 metabolite features
Negative: 6495 metabolite features
Controlling peak quality and removing redundency
Positive: 2637 metabolite features
Negative: 2446 metabolite features
Getting MS2 spectra
Positive: 2234 MS2 spectra
Negative: 2123 MS2 spectra
MS2T library
4491 metabolite features with
3832 tagged with MS2 spectra
