# Supplementary figures and images for: Expanding the Coverage of Metabolic Landscape in Cultivated Rice with Integrated Computational Approaches
Source: Genomics Proteomics Bioinformatics. 2021 Feb 23;20(4):702–14. doi: 10.1016/j.gpb.2020.06.018 (PMC9880819; doi:10.1016/j.gpb.2020.06.018)

**A RSM03724n**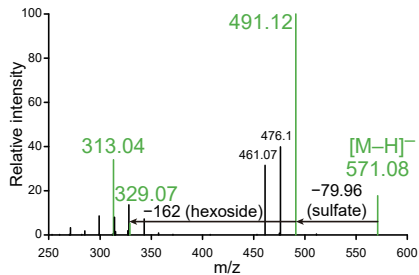**B RSM04661n**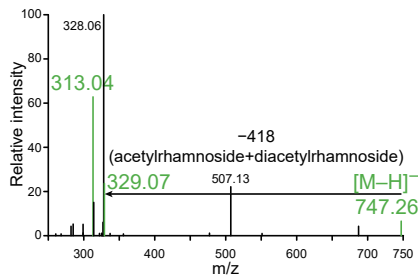**C RSM05814p**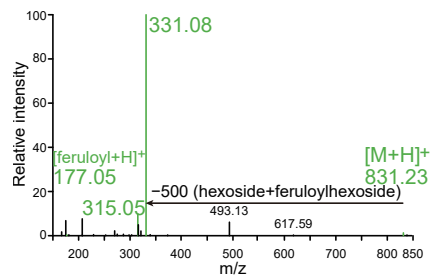**D RSM03991p**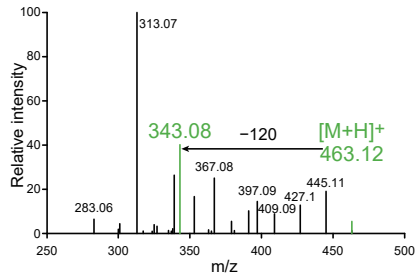**E RSM04767p**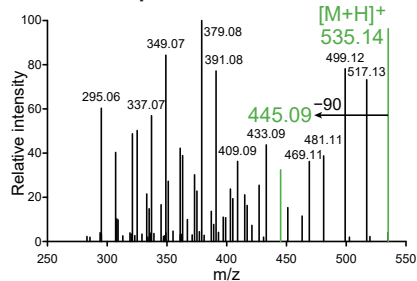

Supplement: Supplementary Figure S3 — The mass spectra of several featured metabolites of indica and japonica cultivars A. The mass spectra of RSM03724n (tricin O-sulfatohexoside). The m/z 313.03586 and 329.06653 are the featured deprotonated ions of tricin, and the neutral losses of m/z 79.95697 and 162.0536 correspond to the sulfate and hexoside groups, respectively. B. The mass spectra of RSM04661n (tricin O-acetylrhamnoside-O-diacetylrhamnoside). The neutral loss of m/z 418.1954 corresponds to the acetylrhamnoside and diacetylrhamnoside groups. C. The mass spectra of RSM05814p (tricin O-feruloylhexoside-O-hexoside). The m/z 315.04944 and 331.08084 are the featured protonated ions of tricin, and the neutral loss of m/z 500.1448 corresponds to the feruloylhexoside and hexoside groups. The m/z 177.05428 is the featured protonated ion of feruloyl unit. D. The mass spectra of RSM03991p (trihydroxy-methoxyflavone C-hexoside). The neutral loss of 120.0449 is the characteristic of C-hexosylflavones. E. The mass spectra of RSM04767p (di-C, C-pentosyl-apigenin). The neutral loss of 90.05359 is the characteristic of C-pentosylflavones. [M+H]+ and [M−H]−, the protonated and deprotonated precursor ion of metabolites, respectively [file mmc3.pdf]

**A** RSM00909n/RSM00910n/RSM00911n

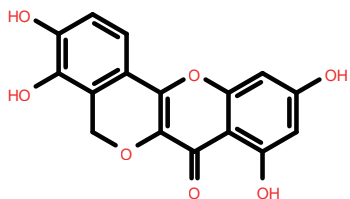

**B** RSM03170p

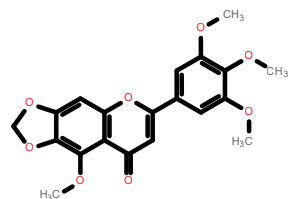

**C** RSM03359p

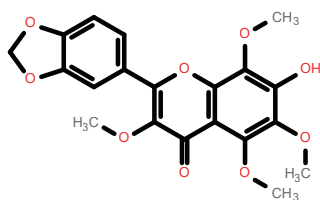

**D** RSM03551p

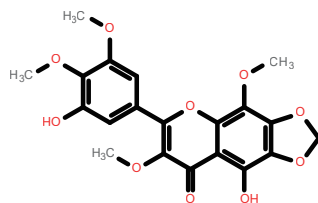

**E** RSM01562p

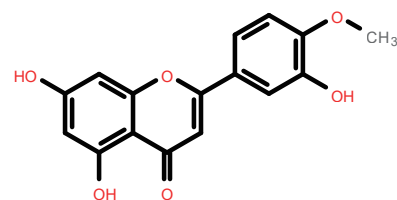

**F** RSM02165p

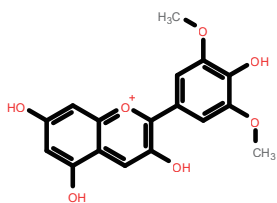

**G** RSM02540p

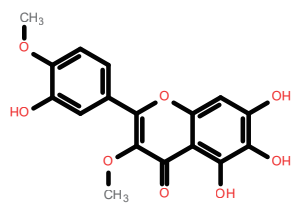

**H** RSM01464n

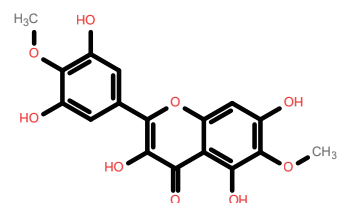

**I** RSM03041p

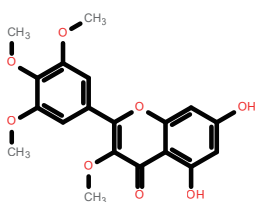

**J** RSM03380p

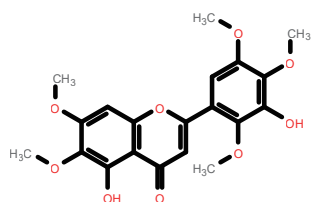

**K** RSM03729p

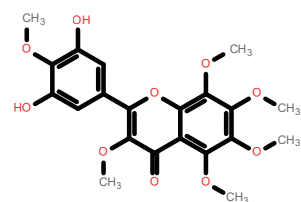

Supplement: Supplementary Figure S5 — The structure of flavonoids within the subgroup of the first-ranked cluster These flavonoids have diverse numbers of hydroxyl and methoxyl groups in their structures [file mmc5.pdf]

**A RSM04382p**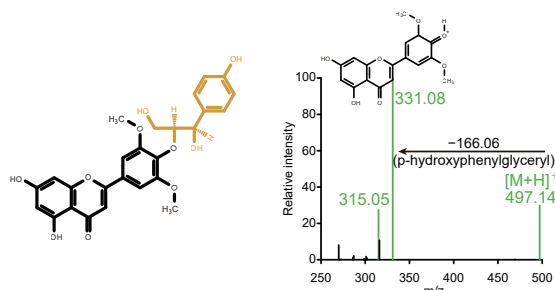**B RSM04355p**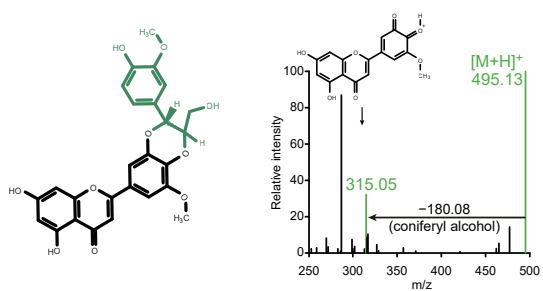**C RSM04702p**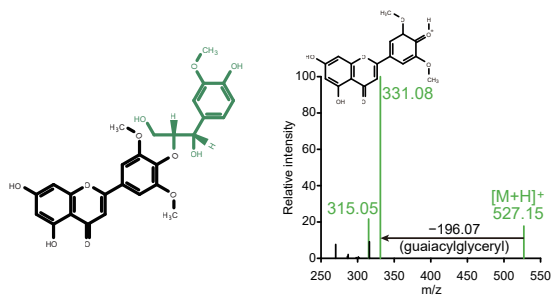**D RSM04691p**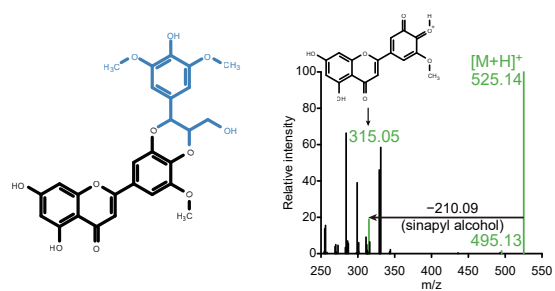**E RSM05479p**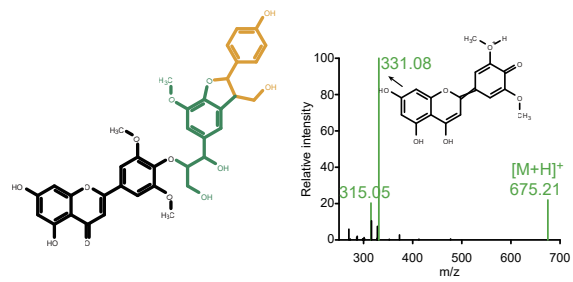**F RSM04546n**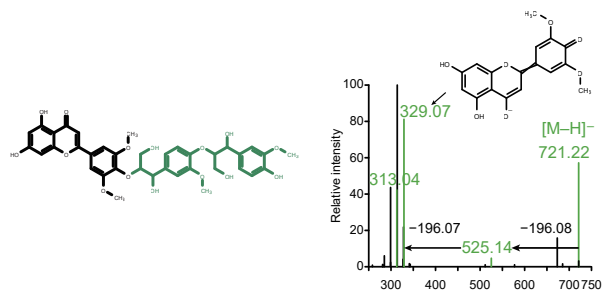**G RSM05574p**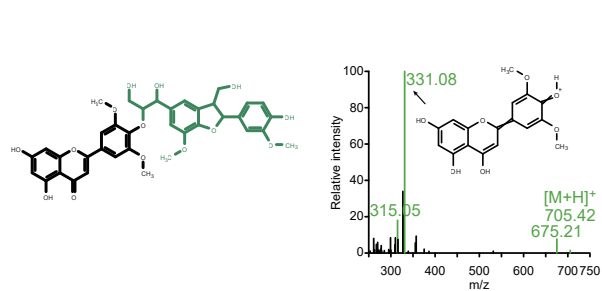**H RSM05474p**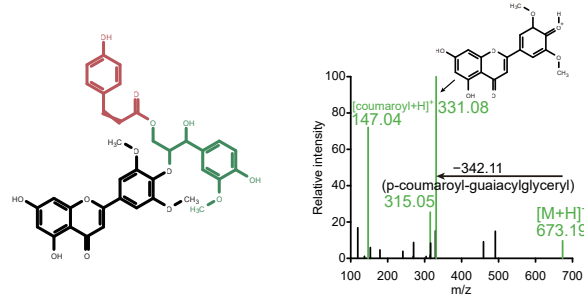**I RSM04164p**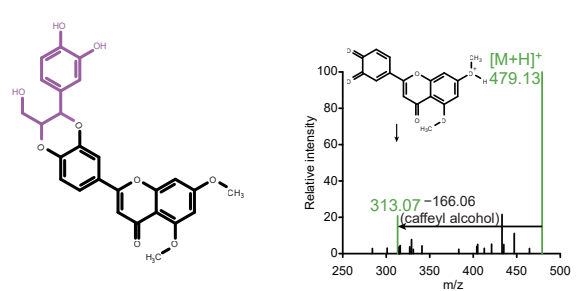**J RSM04201p**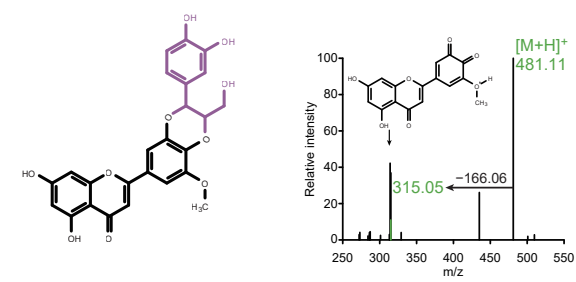

Supplement: Supplementary Figure S6 — The structure and mass spectra of characterized flavonolignans A. RSM04382p, aegicin, which has a structure of tricin moiety with a p-coumaryl alcohol linked by an ether bond. The m/z 315.04950 and 331.08084 are featured protonated ions of tricin, and the neutral loss of m/z 166.0628 corresponds to the p-hydroxyphenylglyceryl unit. B. RSM04355p, 5'-Methoxyhydnocarpin-D, which has a structure of methoxyluteolin moiety with a coniferyl alcohol linked by a dioxane bridge. The m/z 315.04874 is featured protonated ion of methoxyluteolin moiety, and the neutral loss of m/z 180.0789 corresponds to the coniferyl alcohol unit. C. RSM04702p, salcolin B, which has a structure of tricin moiety with a coniferyl alcohol linked by an ether bond. The neutral loss of m/z 196.0736 corresponds to the guaiacylglyceryl unit. D. RSM04691p, palstatin, which has a structure of methoxyluteolin moiety with a sinapyl alcohol linked by a dioxane bridge. The neutral loss of m/z 210.0891 corresponds to the sinapyl alcohol unit. E. RSM05479p, which has a structure of salcolin B moiety with a p-coumaryl alcohol linked by a furan bridge (characterized through its mass spectra). F. RSM04546n, which has a structure of salcolin B moiety with a coniferyl alcohol linked by an ether bond (characterized through its mass spectra). G. RSM05574p, which has a structure of salcolin B moiety with a coniferyl alcohol linked by a furan bridge (characterized through its mass spectra). H. RSM05474p, tricin O-[guaiacyl-(O-p-coumaroyl)-glyceryl] ether, which has an additional coumaroyl unit at the guaiacylglyceryl group of salcolin B. The neutral loss of m/z 342.1071 corresponds to the O-p-coumaroyl-guaiacylglyceryl group, and the m/z 147.04379 is featured protonated ion of p-coumaroyl unit. I. RSM04164p, putative catechyl-type flavonolignans characterized through its mass spectra, which has a structure of dihydroxy-dimethoxyflavone moiety with a caffeyl alcohol linked by a dioxane bridge. The m/z 313.07019 is featur [file mmc6.pdf]

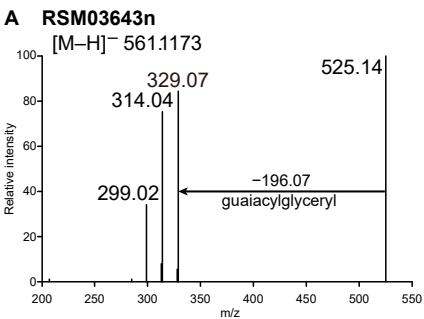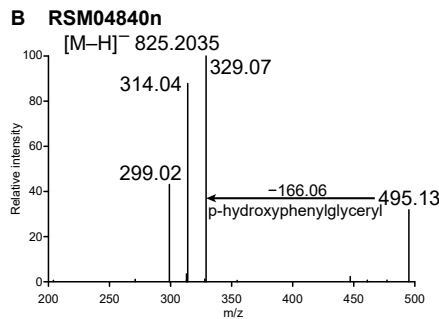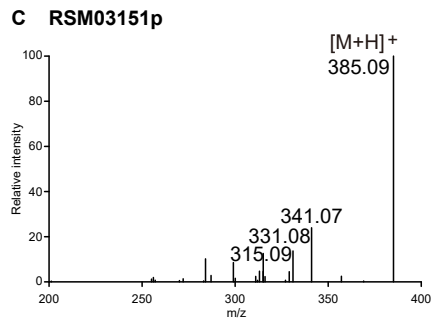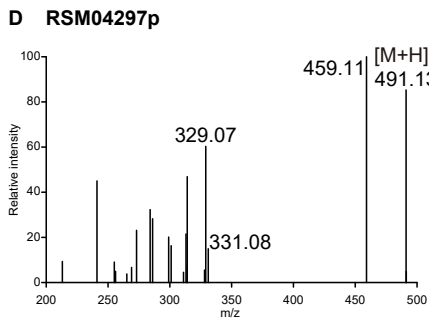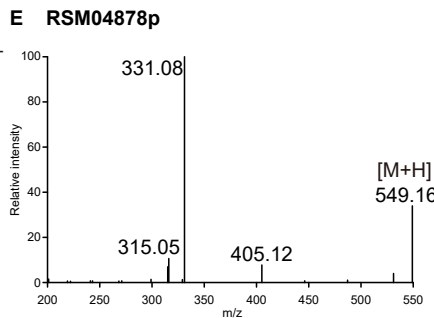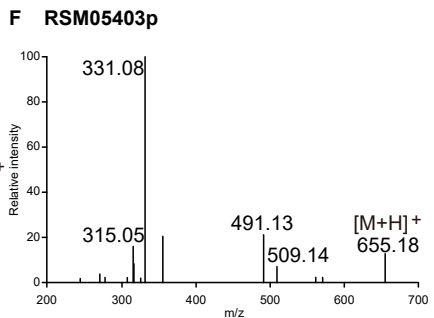

Supplement: Supplementary Figure S7 — The mass spectra of 6 putative tricin derivatives See Table S10 for details [file mmc7.pdf]

**A The first annotation approach evaluation**

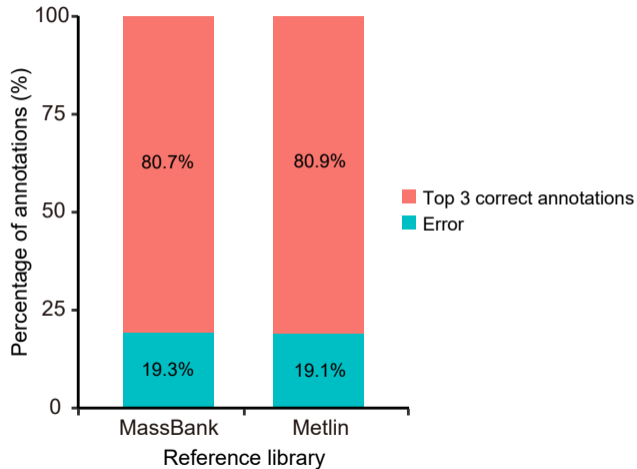

**B The second annotation approach evaluation**

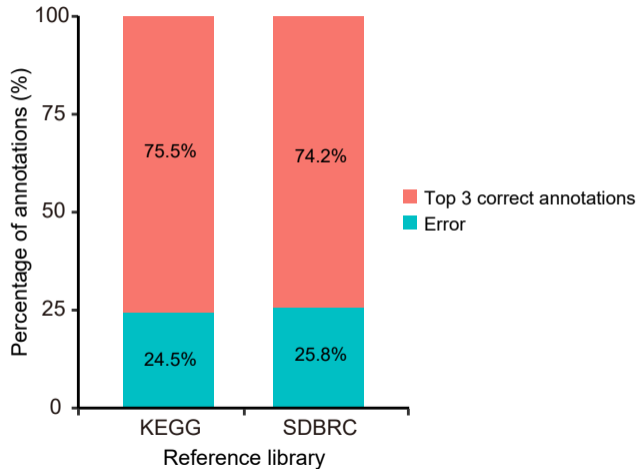

Supplement: Supplementary Figure S8 — The performance evaluation of the annotation approaches with the test set of Fiehn HILIC library The test set used for performance evaluation was collected from the MassBank of North America (see Table S11 for details). A. The percentages of the top 3 correct annotations of the INCOS. The evaluations were performed with Metlin and MassBank as a reference, and the cutoff of the similarity score was 0.75. B. The percentages of the top 3 correct annotations of the second annotation approach. The evaluations were performed with the in silico mass spectra generated from KEGG and SDBRC database as a reference, and the cutoff of the similarity score was 0.3. SDBRC, structural database of biologically relevant compounds [file mmc8.pdf]

**A RSM04010p**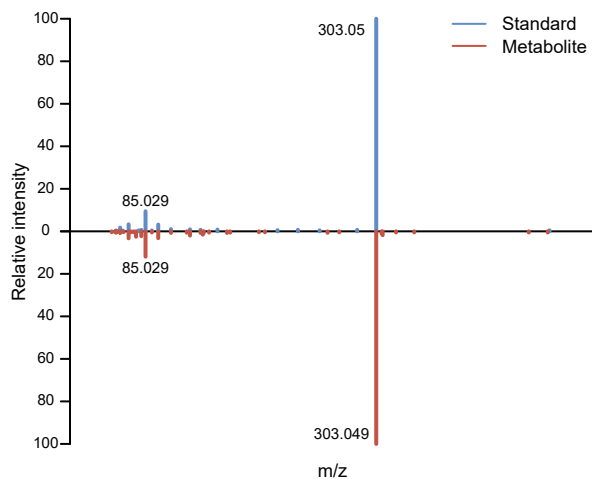**B RSM05067p**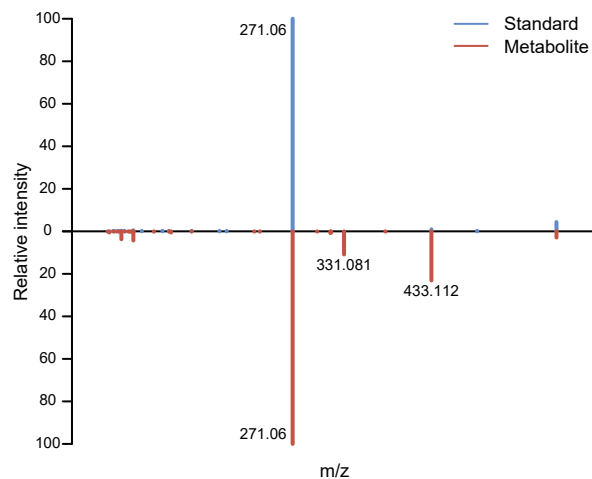**C RSM00535n**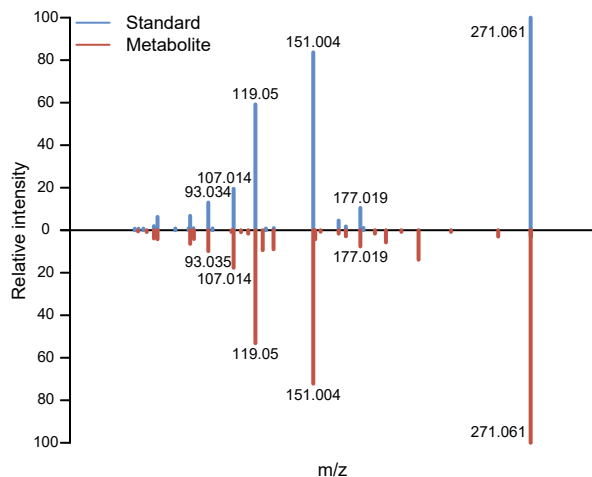**D RSM00117p**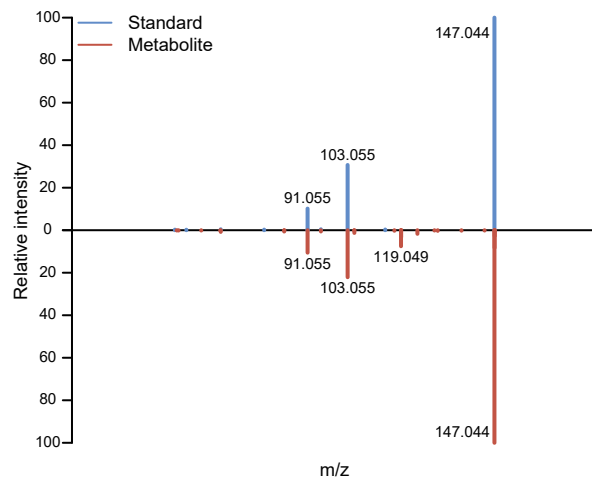**E RSM03072p**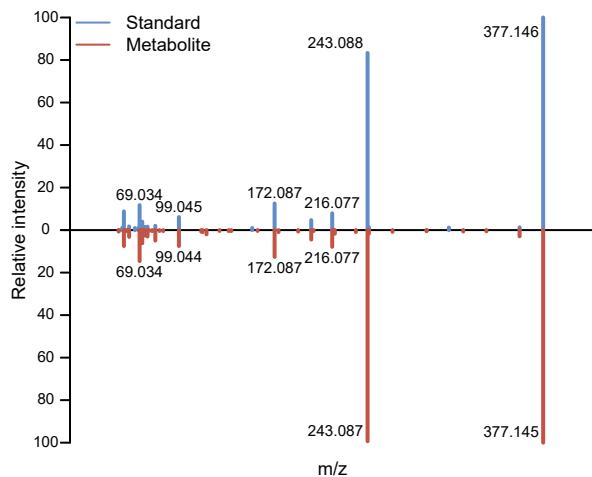**F RSM00290n**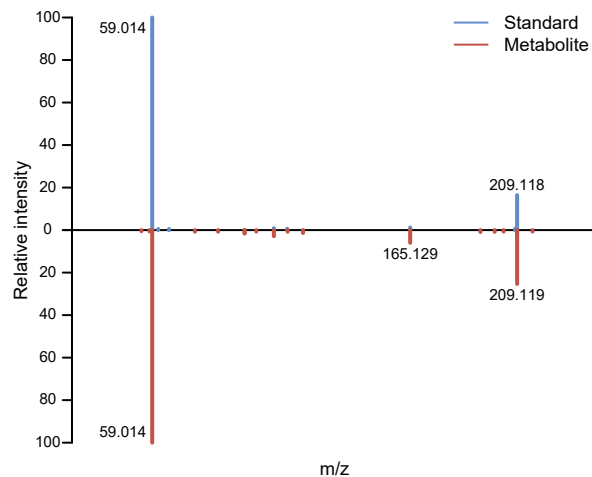**G RSM00089p**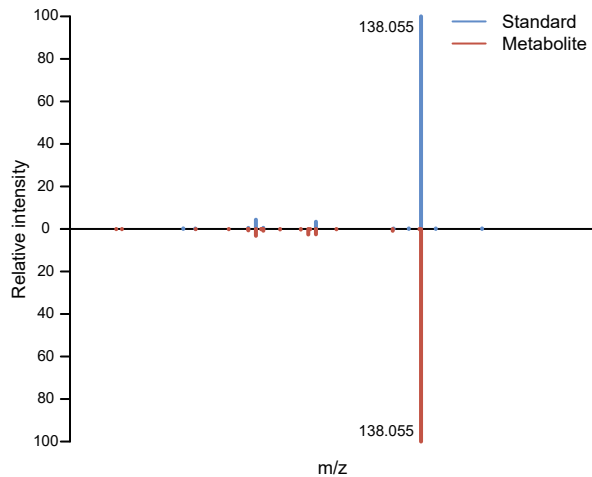**H RSM00234n**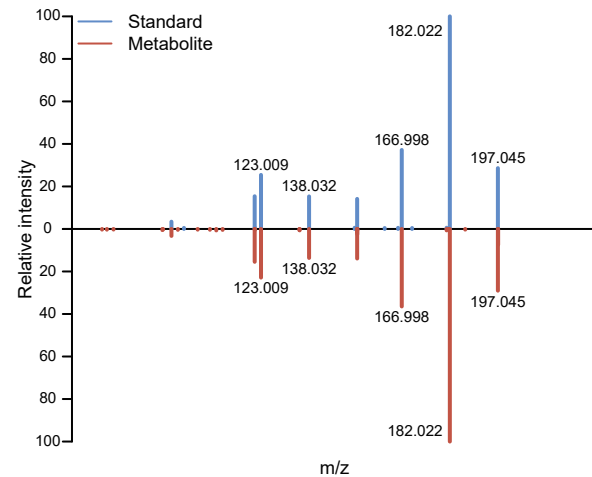

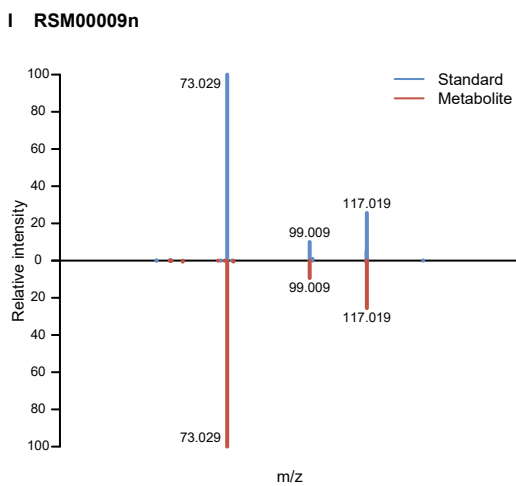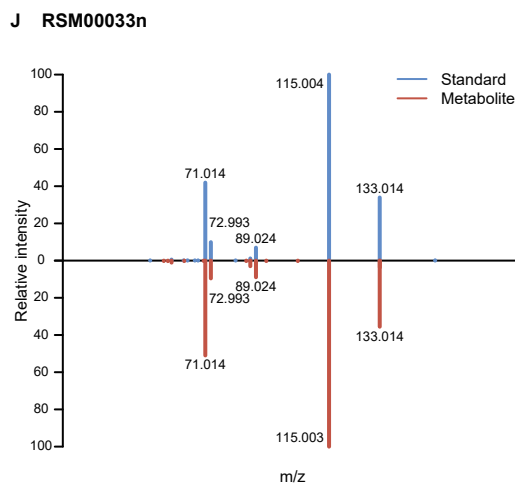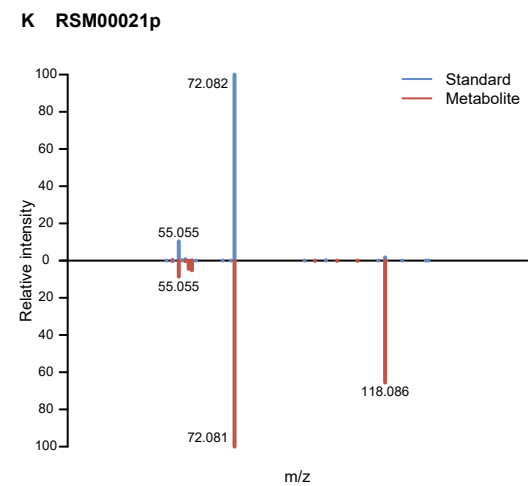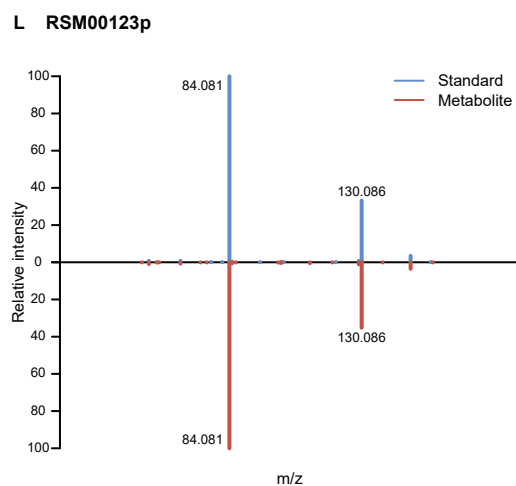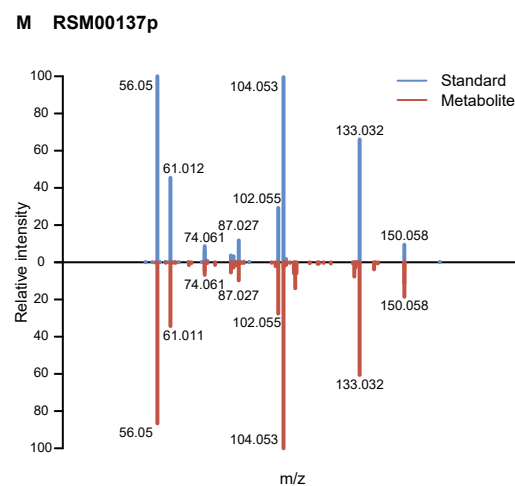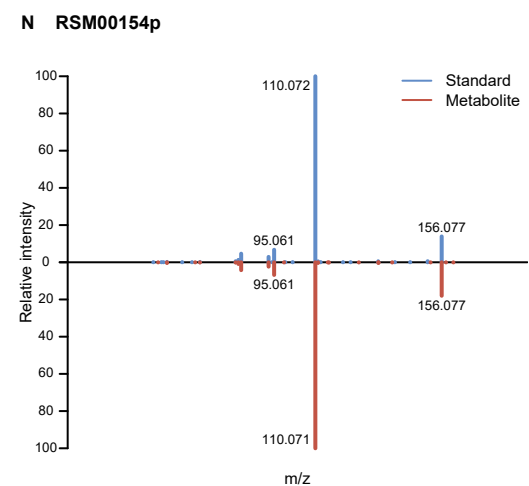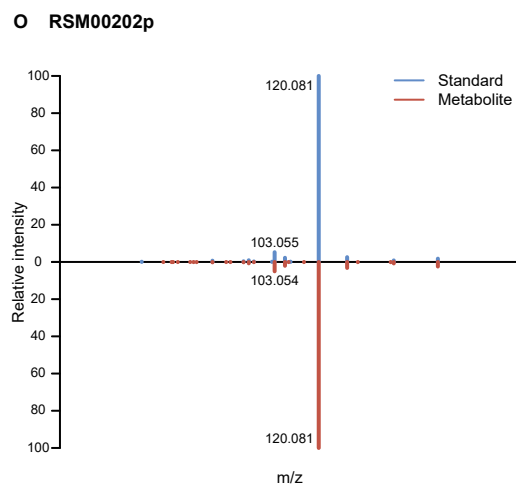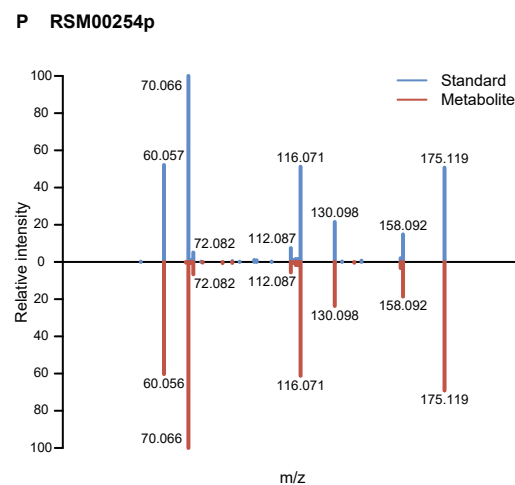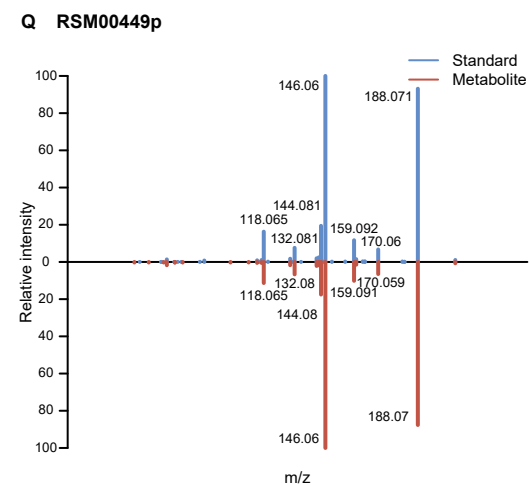

Supplement: Supplementary Figure S9 — The matching results of MS/MS spectra between 17 metabolite features with standard compounds The MS/MS spectra for standard compounds were acquired by the same metabolic analysis methods with metabolite features in the MS2T library. The detailed information for the identification of 17 metabolite features was listed in Table S12 [file mmc9.pdf]
