# Supplementary material for: Expanding the Coverage of Metabolic Landscape in Cultivated Rice with Integrated Computational Approaches
Source: Genomics Proteomics Bioinformatics. 2021 Feb 23;20(4):702–14. doi: 10.1016/j.gpb.2020.06.018 (PMC9880819; doi:10.1016/j.gpb.2020.06.018)

**A The metabolite association network**

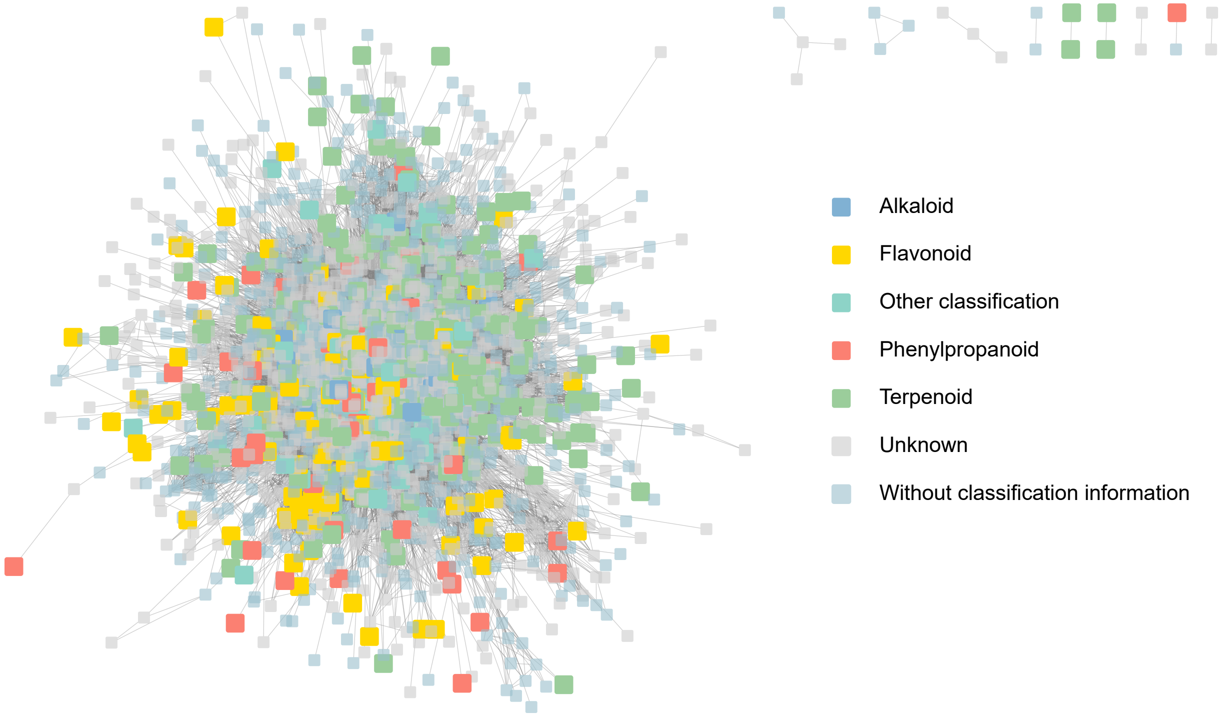


**B The second ranked cluster isolated from the metabolite association network**

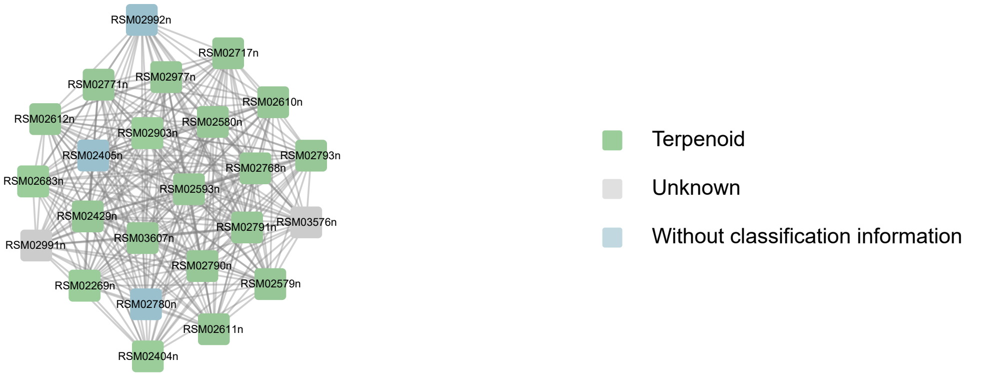

Supplement: Supplementary Figure S4 — The diagram of the metabolite association network A. The metabolite association network of rice grains according to the metabolic profile of 59 rice cultivars. B. The second ranked cluster that mainly consists of terpenoids [file mmc4.pdf]
