# Supplementary material for: Expanding the Coverage of Metabolic Landscape in Cultivated Rice with Integrated Computational Approaches
Source: Genomics Proteomics Bioinformatics. 2021 Feb 23;20(4):702–14. doi: 10.1016/j.gpb.2020.06.018 (PMC9880819; doi:10.1016/j.gpb.2020.06.018)
Supplement: Supplementary Figure S10 — The detailed steps of structural motif search combined with neutral loss scanning (1) The raw structure data of flavones and flavonols were collected from the LIPID MAPS Structure Database. The OpenBabel software was used to convert the raw structure data into the machine-readable structural information, including formula, exact mass, simplified molecular-input line-entry system, and the IUPAC international chemical identifier. The non-redundant structure data for 3145 flavones and flavonols were obtained through merging the compounds with identical structures; (2) The theoretical MS2 spectra for flavones and flavonols were predicted by CFM-ID software with structure data as input; (3) The fragments with the top 25% intensity in each mass spectra were retained for further analysis. The fragments with lower structure similarity (calculated by OpenBabel software) with the diphenylpropane backbone (C6-C3-C6) of flavonoids, were filtered out as non-aglycone derived fragments; (4) The remaining fragments with identical mass were merged and sorted by their frequency. The merged fragments were considered as the characteristic fragments of flavones and flavonols. The fragments with comparatively high frequency represent the structural motifs frequently found in flavones and flavonols, such as m/z at 287.0550145 (featured ion of kaempferol derivatives), m/z at 303.0499291 (featured ion of quercetin derivatives), m/z at 271.0600999 (featured ion of apigenin derivatives), and m/z at 301.0706646 (featured ion of chrysoeriol derivatives), etc.; (5) The mass difference between precursor ion and characteristic fragments in each MS2 spectra were calculated as neutral losses. The neutral losses with similar mass (tolerance: 10 ppm) were merged and sorted by their frequency. The merged neutral losses were considered as the featured neutral losses of flavones and flavonols. The neutral losses with comparatively high frequency represent the modifications frequently occurred in flavones [file mmc10.pdf]

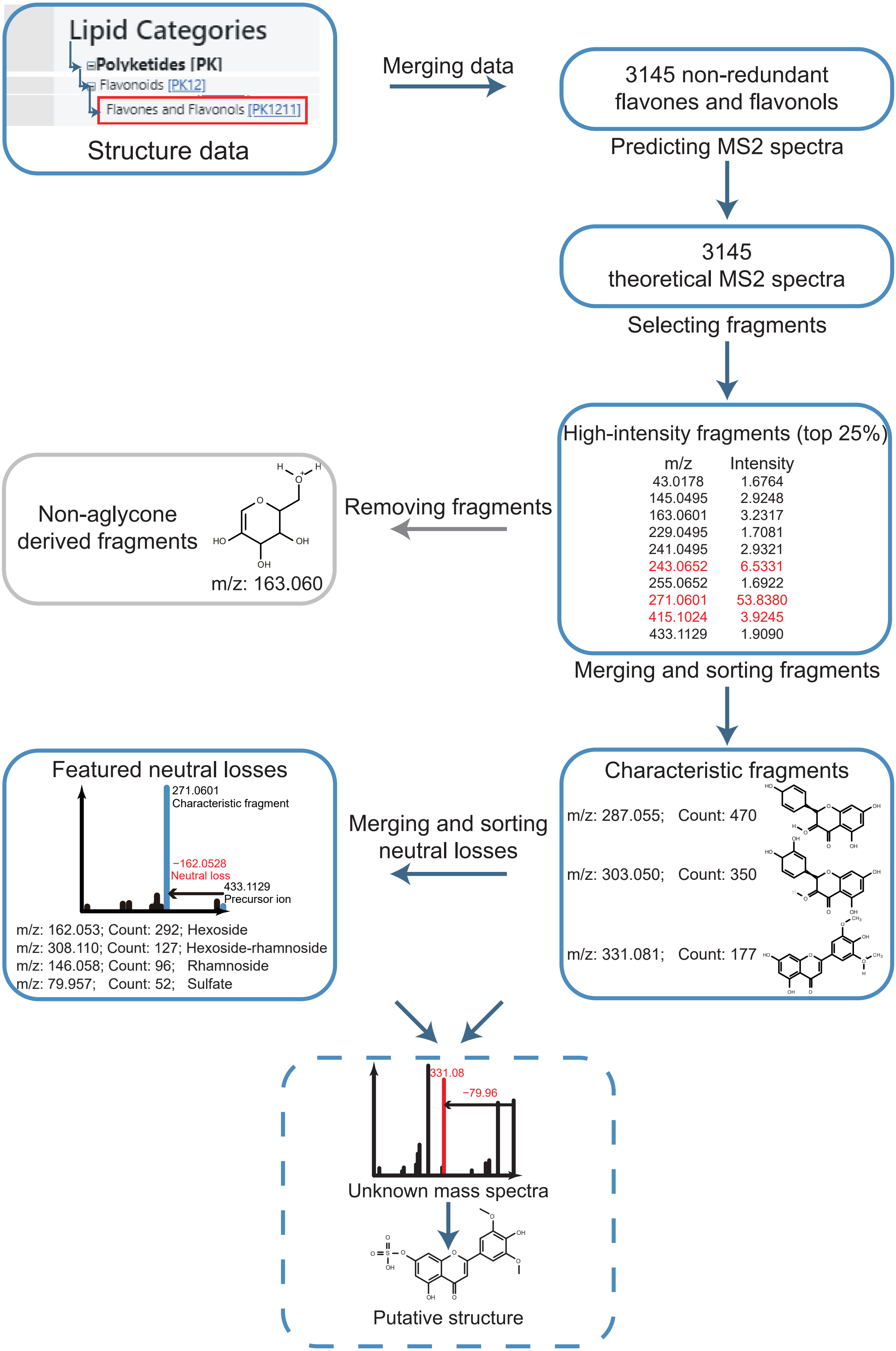

Lipid Categories
Polyketides [PK]
Flavonoids [PK12]
Flavones and Flavonols [PK1211]
Structure data
Merging data
3145 non-redundant flavones and flavonols
Predicting MS2 spectra
3145 theoretical MS2 spectra
Selecting fragments
High-intensity fragments (top 25%)
m/z Intensity
43.0178 1.6764
145.0495 2.9248
163.0601 3.2317
229.0495 1.7081
241.0495 2.9321
243.0652 6.5331
255.0652 1.6922
271.0601 53.8380
415.1024 3.9245
433.1129 1.9090
Removing fragments
Non-aglycone derived fragments
m/z: 163.060
Merging and sorting fragments
Characteristic fragments
m/z: 287.055; Count: 470
m/z: 303.050; Count: 350
m/z: 331.081; Count: 177
Merging and sorting neutral losses
Featured neutral losses
271.0601
Characteristic fragment
−162.0528
Neutral loss
433.1129
Precursor ion
m/z: 162.053; Count: 292; Hexoside
m/z: 308.110; Count: 127; Hexoside-rhamnoside
m/z: 146.058; Count: 96; Rhamnoside
m/z: 79.957; Count: 52; Sulfate
331.08
−79.96
Unknown mass spectra
Putative structure
