# Supplementary material for: Expanding the Coverage of Metabolic Landscape in Cultivated Rice with Integrated Computational Approaches
Source: Genomics Proteomics Bioinformatics. 2021 Feb 23;20(4):702–14. doi: 10.1016/j.gpb.2020.06.018 (PMC9880819; doi:10.1016/j.gpb.2020.06.018)
Supplement: Supplementary File S1 — The detailed steps for the acquisition and processing of relative abundance data of metabolites [file mmc24.docx]

**File S1 The detailed steps for the acquisition and processing of relative abundance data of metabolites**

The raw abundance data were obtained from the peak area of each compound features. The peak area was calculated from raw mass spectrometric data through Compound Discoverer software (v2.0, Thermo Scientific) with its untargeted metabolomics workflow. Through this way, we obtained a matrix of metabolic profiles containing the abundance data of compound features for 59 diverse rice varieties.

The compound features in the matrix of metabolic profiles were aligned with our MS2T library based on accurate mass (tolerance: 5ppm) and retention time (tolerance: 0.35min), for obtaining the corresponding structural information.

A series of quality control methods were applied to reduce the analytical errors caused by artificial factors in the sample preparation and instrumental drift in detection processes as described by previous study (see "Analytical Error Reduction Using Single Point Calibration for Accurate and Precise Metabolomic Phenotyping" for details). First, the abundance data of pre-added internal standard was used to normalize the data of other compound features. $X_{p,i}$, abundance of peak *p* (compound feature) for sample *i*; $X_{is,i}$, abundance of peak *is* (internal standard) for sample *i*; $X_{p,i}^{'}$, relative abundance of peak *p* after the internal standard normalization. The abundance $X_{p,i}$ was normalized into the relative abundance $X_{p,i}^{'}$ using the abundance$X_{is,i}$, as denoted in Equation 1. The multiplication by ${10}^{7}$ (the order of magnitude of the abundance of internal standard) could maintain the initial order of magnitude of the abundance of compound features and avoid the negative values in subsequent log2-transformation.

$X_{p,i}^{'}=\frac{X_{p,i}}{X_{is,i}}\times{10}^{7}$ (1)

Second, the reference control mixture samples were inserted into the analytical sequence once every 10 samples to monitor the performance of instrument. The systematic differences between different batches were corrected by the average value of the reference control mixture samples within each batch (once every 10 samples). The batch 1 was taken as a reference to correct the systematic differences of other batches. $<X_{p,rcm,1}^{'}>$, the average (< >) of relative abundance per peak of reference control mixture (*rcm*) samples in batch 1 after the internal standard normalization; $<X_{p,rcm,b}^{'}>$, the average (< >) of relative abundance per peak of *rcm* samples per batch after the internal standard normalization; $X_{p,b,i}^{'}$, relative abundance of peak *p* for sample *i* per batch after the internal standard normalization; $X_{p,b,i}^{''}$, relative abundance of peak *p* for sample *i* per batch after the reference control mixture samples correction. The systematic differences within different batches were corrected by the multiplication by the ratio of $<X_{p,rcm,1}^{'}>$ and $<X_{p,rcm,b}^{'}>$ (Equation 2).

$X_{p,b,i}^{''}=\frac{<X_{p,rcm,1}^{'}>}{<X_{p,rcm,b}^{'}>}X_{p,b,i}^{'}$ (2)

The normalized relative abundance data of two biological repeats per rice accession was averaged and log2-transformed for subsequent analysis.
